# LINC01232 Promotes Gastric Cancer Proliferation through Interacting with EZH2 to Inhibit the Transcription of KLF2

**DOI:** 10.4014/jmb.2106.06041

**Published:** 2021-08-19

**Authors:** Jing Liu, Zhen Li, Guohua Yu, Ting Wang, Guimei Qu, Yunhui Wang

**Affiliations:** 1 Department of Pathology, Yantai Yuhuangding Hospital, Yantai 264000, P.R. China; 2 Department of General and Pediatric Surgery, Yantai Yuhuangding Hospital, Yantai 264000, P.R. China

**Keywords:** LINC01232, EZH2, KLF2, gastric cancer, proliferation

## Abstract

To clarify the role of long intergenic nonprotein-coding RNA 1232 (LINC01232) in the progression of gastric cancer and the potential mechanism, we analyzed the expression of LINC01232 in TCGA database using the GEPIA online tool, and the LINC01232 level in gastric cancer cell lines was detected by quantitative real time-polymerase chain reaction (qRT-PCR) as well. Cell proliferation assay, colony formation assay, transwell assay and tumor formation experiment in nude mice were conducted to observe the biological behavior changes of gastric cancer cells through the influence of LINC01232 knockdown. LncATLAS database and subcellular isolation assay were used for subcellular distribution of LINC01232 in gastric cancer cells. The interaction among LINC01232, zeste homolog 2 (EZH2) and kruppel-like factor 2 (KLF2) was clarified by RNA-protein interaction prediction (RPISeq), RNA immunoprecipitation (RIP), qRT-PCR and chromatin immunoprecipitation (ChIP) assay. Rescue experiments were further conducted to elucidate the biological function of LINC01232/KLF2 axis in the progression of gastric cancer. LINC01232 was upregulated in stomach adenocarcinoma (STAD) tissues and gastric cancer lines. LINC01232 knockdown inhibited the proliferative capacities of gastric cancer cells in vitro, and impaired in vivo tumorigenicity. LINC01232 was mainly distributed in the cell nucleus where it epigenetically repressed KLF2 expression via binding to the enhancer of EZH2, which was capable of binding to promoter regions of KLF2 to induce histone H3 lysine 27 trimethylation (H3K27me3). LINC01232 exerts oncogenic activities in gastric cancer via inhibition of KLF2, and therefore, the knockdown of KLF2 could reverse the regulatory effect of LINC01232 in the proliferative ability of gastric cancer cells.

## Introduction

Gastric cancers form malignant tumors originating from the gastric mucosal epithelium, and they rank fourth and second in the incidence and mortality of malignant tumors respectively [1]. Each year about 990,000 people are diagnosed with gastric cancer globally and about 738,000 of them die of the disease [2]. Because there are no obvious symptoms in the early clinical stage, the early diagnosis rate of gastric cancer is low. Surgery is the only chance of cure, but recurrence is common, even with complete removal [3]. Gastric cancer poses a serious health burden worldwide, thus, clarifying the molecular mechanism of the occurrence and development of gastric cancer is conducive to finding new therapeutic targets and improving the prognosis of patients with this disease.

Long non-coding RNAs (lncRNAs) are a type of RNA with a length of over 200 nucleotides that are incapable of coding proteins. A number of studies have confirmed that lncRNAs are involved in the regulation of tumor growth signals [4-6]. LncRNA has a variety of biological functions in all stages of cancer development, including cell development and immunity, cell proliferation and differentiation, and apoptosis regulation [7]. Previous evidence has indicated that long intergenic nonprotein-coding RNA 1232 (LINC01232) exerts an oncogenic role in numerous types of tumors, including pancreatic adenocarcinoma, esophageal squamous cell carcinoma, *et al*, [8, 9]. In this study, we pre-analyzed the expression of LINC01232 in TCGA database using the GEPIA online tool. The results showed that LINC01232 is upregulated in gastric cancer samples and predicted poor prognosis. Numerous studies have indicated that LINC01232 may be a new potential molecular biomarker for tumor diagnosis and tumor therapy. Thus, we chose LINC01232 for functional and mechanism analysis in gastric cancer cell lines.

Kruppel-like factors (KLFs) are a subclass of the zinc-finger family of DNA-binding transcription factors, which play a diverse role in various biological processes including cellular growth and differentiation [10]. Sindi HA *et al*. found that reduced KLF2 signaling is a common feature of human pulmonary arterial hypertension and highlights the potential therapeutic role of KLF2-regulated exosomal miRNAs in pulmonary arterial hypertension and other diseases associated with vascular remodeling [11]. Furthermore, the research of Li Q *et al*. showed that lncRNAs can interact with RNA-binding proteins (RBPs) to induce gene expression regulation at the transcriptional level [12]. Through bioinformatics analysis, we found that EZH2 was predicted to be an RBP that could bind LINC01232. Therefore, in this study we investigated the effects of LINC01232 targeting KLF2 on the proliferation of gastric cancer cells, which may potentially be used as a therapeutic target for the treatment of gastric cancer in future.

## Materials and Methods

### Cell Culture and Transfection

Human gastric mucosal cell GES-1 and human gastric cancer cells AGS, SGC-7901, MKN-45, MKN-28, and HGC-27 were purchased from Punosai Life Science and Technology Co., Ltd. (China). AGS cells were cultured in Ham's F-12 medium (Gibco, Invitrogen, USA) containing 10% fetal bovine serum (FBS, Solarbio, China), 100 μg/ml streptomycin and 100 U/ml penicillin. GES-1, SGC-7901, MKN-45, MKN-28 and HGC-27 cells were cultured in RPMI 1640 medium (Gibco, Invitrogen) containing 10% FBS, 100 μg/ml streptomycin and 100 U/ml penicillin. All cells were cultured at 37°C, 5% CO_2_, and saturated humidity.

Cell transfection was performed by using Lipofectamine 3000 transfection reagent (Thermo Fisher Scientific, USA) based on the manufacturer's instructions. ShRNA sequences used in this study were as follows: sh-NC: CCGCGGACTTGCCTCCTACACTACTCHAGTAGTGTAGGAGGCAAGTCCTTTTTG; sh-LINC01232#1: CCG CGACACGTCATCTAGAATAACTCHAGTTATTCTAGATGACGTGTCTTTTTG; sh-LINC01232#2: CCGCCA AGAGTGCTGGATCTAAACTCHAGTTTAGATCCAGCACTCTTGTTTTTG; sh-LINC01232#3: CCGCGA TTGGTTGCTTTCTGCAACTCHAGTTG CAGAAAGCAACCAATCTTTTTG; sh-KLF2: CACCGCAAG ACCTACACCAAGAGTTCGAAAACTCTTGGTGTAGGTCTTG. si-EZH2: 5'-GACUCUGAAUGCAGUUGC UTT-3'; si-NC: 5'-UUCUCCGAACGUGUCACGUTT-3'.

### Bioinformatics Analysis

The expression of lncRNA was analyzed by GEPIA, http://gepia2.cancer-pku.cn/#index. The RBP was predicted by RPISe, http://pridb.gdcb.iastate.edu/RPISeq/#. The lncRNA subcellular localization was predicted by lcnatlas, lncrnahttps://lncatlas.crg.eu/.

### Quantitative Real-Time Polymerase Chain Reaction (qRT-PCR)

Total RNA was extracted by TRIzol Reagent (Thermo Fisher Scientific, USA) and RNA was then transcribed into cDNA using SuperScript III Reverse Transcriptase (Baosheng Scientific, China). QRT-PCR was performed using the SYBR Green PCR Master Mix (Takara, Japan). The experiment was repeated for three times and the relative expression level was calculated by 2^−ΔΔCT^ method and using GAPDH or U6 expression as normalizing control. The primer sequences were as follows: LINC01232: 5′-AGGATGCGCCTAAGAAAGGG-3′ (F) and 5′-CCGGGGGATTGAGGAAACAT-3′ (R); p21: 5′-TGTCCGTCAGAACCCATGC-3′ (F) and 5′-AAAGTCGAA GTTCCATCGCTC-3′ (R); p53: 5′-CAGCACATGACGGAGGTTGT-3′ (F) and 5′-TCATCCAAATACTCC ACACGC-3 (R); KLF2: 5′-CTGCACATGAAACGGCACAT-3′ (F) and 5′-CAGTCACAGTTTGGGAGGGG-3′(R); LATS2: 5′-ACCCCAAAGTTCGGACCTTAT-3′ (F) and 5′-CATTTGCCGGTTCACTTCTGC-3′ (R); EZH2: 5′-AATCAGAGTACATGCGACTGAGA-3′ (F) and 5′-GCTGTATCCTTCGCTGTTTCC-3′ (R); GAPDH: 5′-GGACACAATGGATTGCAAGG-3′ (F) and 5′-TAACCACTGCTCCACTCTGG-3′ (R); U6: 5′-CTCGCTTCGGCAGCACA-3′ (F) and 5′-AACGCTCACGAATTTGCGT-3′ (R).

### MTT Assay

Logarithmic growth cells were cultured in 96-well plates at 37°C with a density of 2,000 cells/well. After 0, 24, 48, 72, and 96 h, 5 mg/ml MTT (Japan) was added to the cells and incubation continued for another 2 h. Then, the medium was removed and 200 μl DMSO was added to dissolve the formazan crystal. The optical density (OD) was measured by microplate reader (BioRad, USA) at 490 nm.

### Colony Formation Assay

Next, 1,000 cells/well were planted in 12-well plates and cultured for 14 days, with the medium being changed every 3 days. The colonies were then fixed with paraformaldehyde and stained with crystal violet, and photographs were taken to calculate the number of colonies formed.

### Transwell Assay

After the precipitated cells were resuspended, the cell density was adjusted to 1×10^6^ cells/ml in serum-free medium. Next, 100 μl cell suspension was added into the upper compartment of the chamber and incubated in a cell incubator for 10 min. For cell invasion, 50 μl matrix gel was precoated on the membrane in the Transwell chamber and cured in an incubator at 37°C for 30 min. Then, 100 μl cell solution was added to the surface of the matrix gel. Following this, 600 ul of complete medium containing 10% FBS was added to the lower chamber. After 24 h incubation, the migrating or invaded cells were fixed with methanol, stained with 1% crystal violet (Solarbio, China) for 10 min and observed under a light microscope (Olympus Corp., Japan) at ×200 magnification.

### Subcellular Isolation Assay

RNA was isolated from the cytoplasm or nucleus using cytoplasmic and nuclear RNA purification kits (Norgen, Canada). The collected cells were lysed on ice and centrifuged at 12,000 g for 3 min. The RNA levels in the cytoplasm and nucleus of the supernatant were analyzed using U6 as nuclear control and GAPDH as cytoplasmic control.

### RNA Immunoprecipitation (RIP)

RIP assay was performed using the Magna RIP RNA-Binding Protein Immunoprecipitation Kit (Millipore Corp., USA). Cell extracts were harvested with RIP lysis buffer and mixed with magnetic beads and antibodies against EZH2, LSD1 and SUZ12 (Abcam, UK). With anti-IgG antibody as control, the co-precipitated RNA was purified and analyzed by qRT-PCR.

### Chromatin Immunoprecipitation (ChIP) Assay

Transferred cells were collected and fixed with 4% PFA for 15 min. After removing the fixation solution, the cells were incubated with 0.65M glycine solution under mild agitation for 5 min. The cells were fixed with PBS and lysed in ChIP lysis buffer on ice for 15 min. Then, the lysates were ultrasonized and the chromatin DNA was cut into 200-800 bp fragments. After centrifugation, the supernatant was collected and the chromatin fragments were incubated overnight at 4°C with anti-EZH2 (AB195409, Abcam), anti-H3K27Me3 (AB192985, Abcam) or recombinant rabbit IgG (AB172730, Abcam). Protein agarose/agarose was used to precipitate endogenous DNA-protein complexes. Finally, the precipitate was decross-linked overnight at 65°C, and the DNA fragments were purified and extracted with phenol/chloroform. Input (partial DNA fragment) was used as the internal control. The sequence of ChIP primers was as follows: KLF2: 5'-CCTCAGTTTCCCTGCACTTGAC-3' (F) and 5'-GAGATACAATCACACCACTAC-3' (R).

### Western Blot Analysis

Cells were lysed in RIPA buffer (Beyotime Institute of Biotechnology, China) and the protein concentration was measured with bicinchoninic acid (Pierce BCA Protein Assay Kit; Thermo Fisher Scientific, Inc.). After electrophoresis on 8% sodium dodecyl sulfate-polyacrylamide gel electrophoresis, protein extract was transferred to a polyvinylidene difluoride membrane and blocked with 3% (w/v) skim milk. Mouse anti-KLF2 antibody (AB194486, 1:1000, Abcam) or mouse anti-GAPDH antibody (AB8245, 1:1000, Abcam) were used as primary antibodies and horseradish peroxidase-conjugated goat anti-mouse IgG (AB205719, 1:2000, Abcam) was used as the secondary antibody. The signals were detected by enhanced chemiluminescence method with GAPDH as an internal control.

### Tumor Formation Experiment in Nude Mice

SPF Balb/c female nude mice (4 weeks old, 16-18 g weight) were purchased from the National Laboratory Animal Center (China). All animal experiments were conducted in accordance with the National Institutes of Health guidelines (Pub. No. 85-23, revised 1996) and all the procedures were reviewed and approved by Yantai Yuhuangding Hospital.

A total of 2×10^6^ HGC-27 cells were inoculated into the soft skin of the right rear forelimb of nude mice and the tumor volume was calculated every 7 days. Tumor volume = (long diameter × short diameter^2^)/2. Four weeks later, the nude mice were anesthetized by intraperitoneal injection of 3% sodium pentobarbital (40 mg/kg body weight) and sacrificed by cervical dislocation, and the cancerous tissue was removed and weighed.

### Statistical Methods

Data were presented as means ± SD and analyzed using SPSS19.0 statistical software (SPSS Inc., USA). The differences between the two groups were analyzed using Student's *t*-test and one-way analysis of variance was used to analysis the differences between multiple groups. *p* < 0.05 was regarded as statistically significant.

## Results

### The Expression of LINC01232 in Stomach Adenocarcinoma (STAD) Tissues and Gastric Cancer Cells

**Fig. 1:**
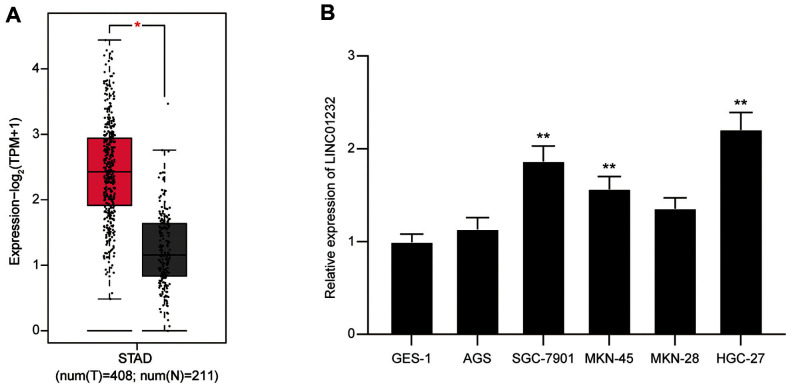
LINC01232 was highly expressed in stomach adenocarcinoma (STAD) tissues and gastric cancer cells. **A**: The expression of LINC01232 in TCGA gastric cancer samples and normal samples. **B**: The expression of LINC01232 in normal cell line (GES-1) and gastric cancer cell lines (AGS, SGC-7901, MKN-45, MKN-28, and HGC-27). Values are mean ± SD. **p < 0.01 vs. CES-1 cells.

The expression of LINC01232 in TCGA database was analyzed using the GEPIA online tool, and the results showed that the expression of LINC01232 in STAD tissues was higher than that in normal tissues (*p* < 0.05, Fig. 1A). Meanwhile, AGS, SGC-7901, MKN-45, MKN-28 and HGC-27 cells were used to detect the expression of LINC01232 in human gastric cancer cells. The results revealed that the expression of LINC01232 mRNA in gastric cancer cells was significantly higher than that in human gastric mucosal cell GES-1 (*p* < 0.05, respectively, Fig. 1B). The above results indicated that the expression of LINC01232 was upregulated in gastric cancer. SGC-7901 and HGC-27 cells with high LINC01232 expression were selected for subsequent functional verification.

### Silencing of LINC01232 Inhibits the Progression of Gastric Cancer

**Fig. 2:**
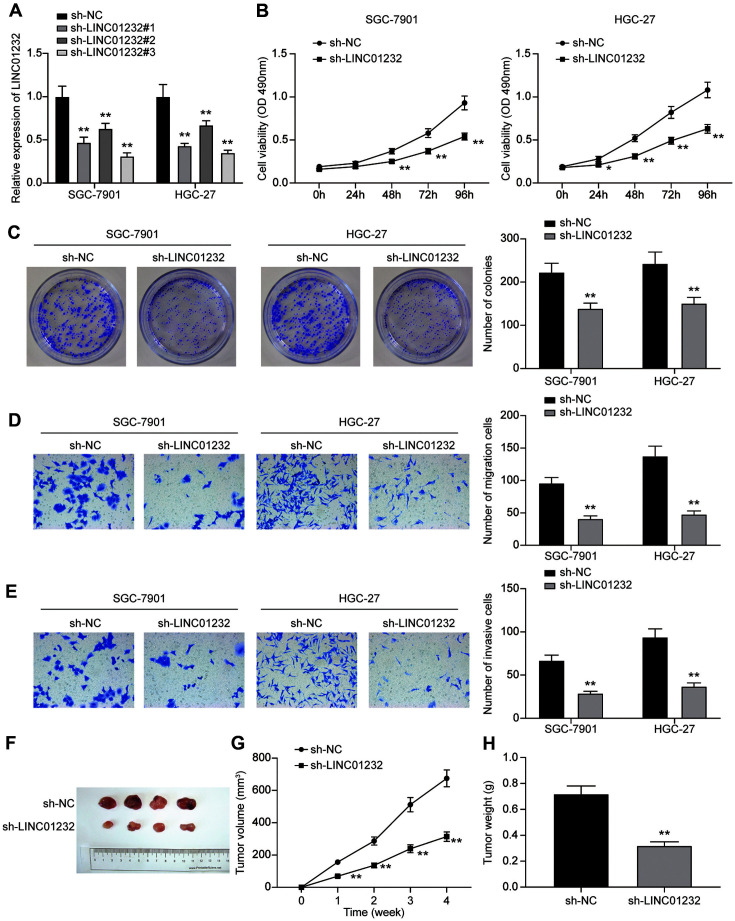
Silencing of LINC01232 inhibits the progression of gastric cancer. **A**: QRT-PCR was used to detect the knockdown efficiency of LINC01232 in SGC-7901 and HGC-27 cells. **B**: MTT assay was used to detect the viability of SGC- 7901 and HGC-27 cells after transfection with sh-NC or sh-LINC01232. **C**: Colony formation assay was used to detect the proliferation of SGC-7901 and HGC-27 cells after transfection with sh-NC or sh-LINC01232. **D, E**: Transwell assay was used to detect the migration and invasion abilities of SGC-7901 and HGC-27 cells after transfection with sh-NC or sh-LINC01232. **F:** Representative xenograft tumor photograph of mice subcutaneously injected with sh-NC or sh-LINC01232 cells. G: Tumor volume. H: Tumor weight. Values are mean ± SD. *p < 0.05, **p < 0.01 vs. sh-NC group.

Three shRNAs and 1 sh-NC targeting LINC01232 were designed and transfected into SGC-7901 and HGC-27 cells, respectively. QRT-PCR showed that the mRNA expression of LINC01232 was significantly reduced by those 3 shRNAs, and sh-LINC01232#3 had the highest knockdown efficiency (Fig. 2A). Therefore, sh-LINC01232#3 was used in the following study. Comparing with the sh-NC group, downregulation of LINC01232 was found to cause a dramatic decrease of cell viability (Fig. 2B), colony formation (Fig. 2C), migration (Fig. 2D) and invasion (Fig. 2E) (*p* < 0.05, respectively). Moreover, a significant decrease of tumor volume and weight was noted in the sh-LINC01232 group compared with that of the sh-NC group (Fig. 2F-H, *p* < 0.05, respectively). These findings demonstrated that LINC01232 knockdown inhibited proliferative capacities of gastric cancer cells in vitro, and impaired in vivo tumorigenicity.

### LINC01232 Inhibits KLF2 Expression to Promote Gastric Cancer Proliferation through EZH2-Mediated H3K27me3

**Fig. 3:**
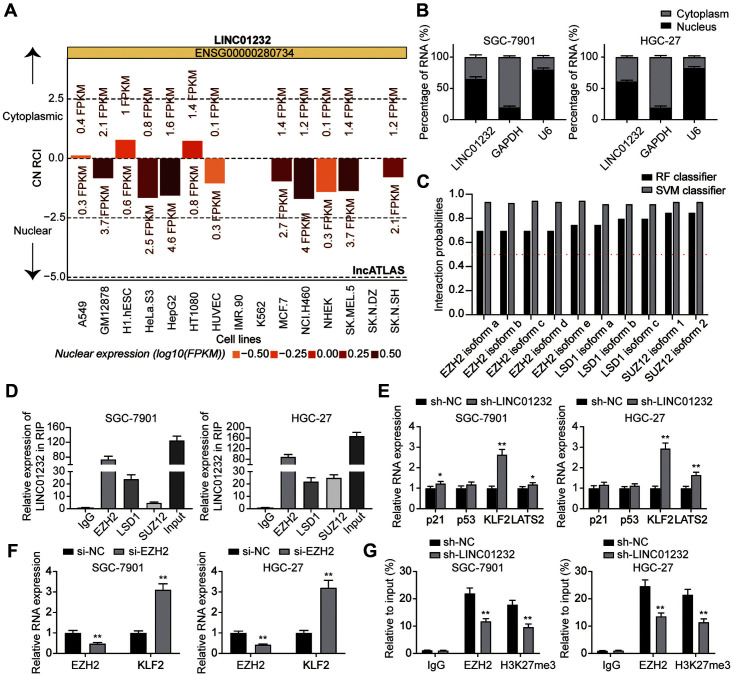
LINC01232 inhibits KLF2 expression to promote gastric cancer proliferation through EZH2- mediated H3K27me3. **A**: LncATLAS database analysis was used to predict the subcellular localization. **B**: Subcellular isolation assay was used to detect the subcellular localization of LINC01232 in cytoplasmic and nuclear sites of SGC-7901 and HGC-27 cells. **C**: RPISeq analysis was used to predict the interaction probability of LINC01232 with EZH2, LSD1 and SUZ12 (The RF and SVM >0.5 was considered to have the ability of binding). **D**: RIP assay was used to detect the interactions of EZH2, LSD1 and SUZ12 with LINC01232 in SGC-7901 and HGC-27 cells. **E**: QRT-PCR was used to detect the expression of P21, p53, KLF2 and LATS2 in SGC-7901 and HGC-27 cells after transfection with sh-NC or sh-LINC01232. **F**: QRT-PCR was used to detect the expression of EZH2?KLF2 in SGC-7901 and HGC-27 cells after transfection with si-NC or si-EZH2. **G**: ChIP assay showed that EZH2 and H3K27me3 were binding to the promoter regions of KLF2. Values are mean ± SD. **p < 0.01 vs. sh-NC group.

LncATLAS database (Fig. 3A) and subcellular isolation assay (Fig. 3B) indicated that LINC01232 was mainly distributed in the cell nucleus. Through bioinformatics analysis, we found that EZH2, LSD1 and SUZ12 are the RBPs that could bind LINC01232 (Fig. 3C). RIP assay revealed that LINC01232 was in the immunoprecipitation of EZH2 and LSD1 from the lysates of SGC-7901 and HGC-27 cells (Fig. 3D). LINC01232 is highly expressed in EZH2 immunoprecipitation, so we focused on the interaction between LINC01232 and EZH2. QRT-PCR showed that LINC01232 knockdown had the greatest effect on the mRNA expression of KLF2 and LATS2 (Fig. 3E). Because EZH2 had the most obvious effect on KLF2, we focused on the relation between EZH2 and KLF2. Further study indicated that the decrease of EZH2 expression can promote the mRNA expression of KLF2 (Fig. 3F). Previous studies have reported that lncRNA has epigenetic silencing of KLF2 transcription through EZH2-mediated H3K27me3 methylation, and therefore we wondered whether this mechanism exists in LINC01232 [13, 14]. ChIP assay showed that the binding of EZH2 to the KLF2 promoter region was impaired. In addition, the enrichment of H3K27Me3 in the KLF2 promoter region was also decreased after the downregulation of LINC01232 (Fig. 3G). All these results suggested that LINC01232 can directly interact with EZH2 and inhibit the expression of KLF2 through H3K27me3 histone methylation.

### LINC01232 Exerts Oncogenic Activities in Gastric Cancer Via Inhibition of KLF2

**Fig. 4:**
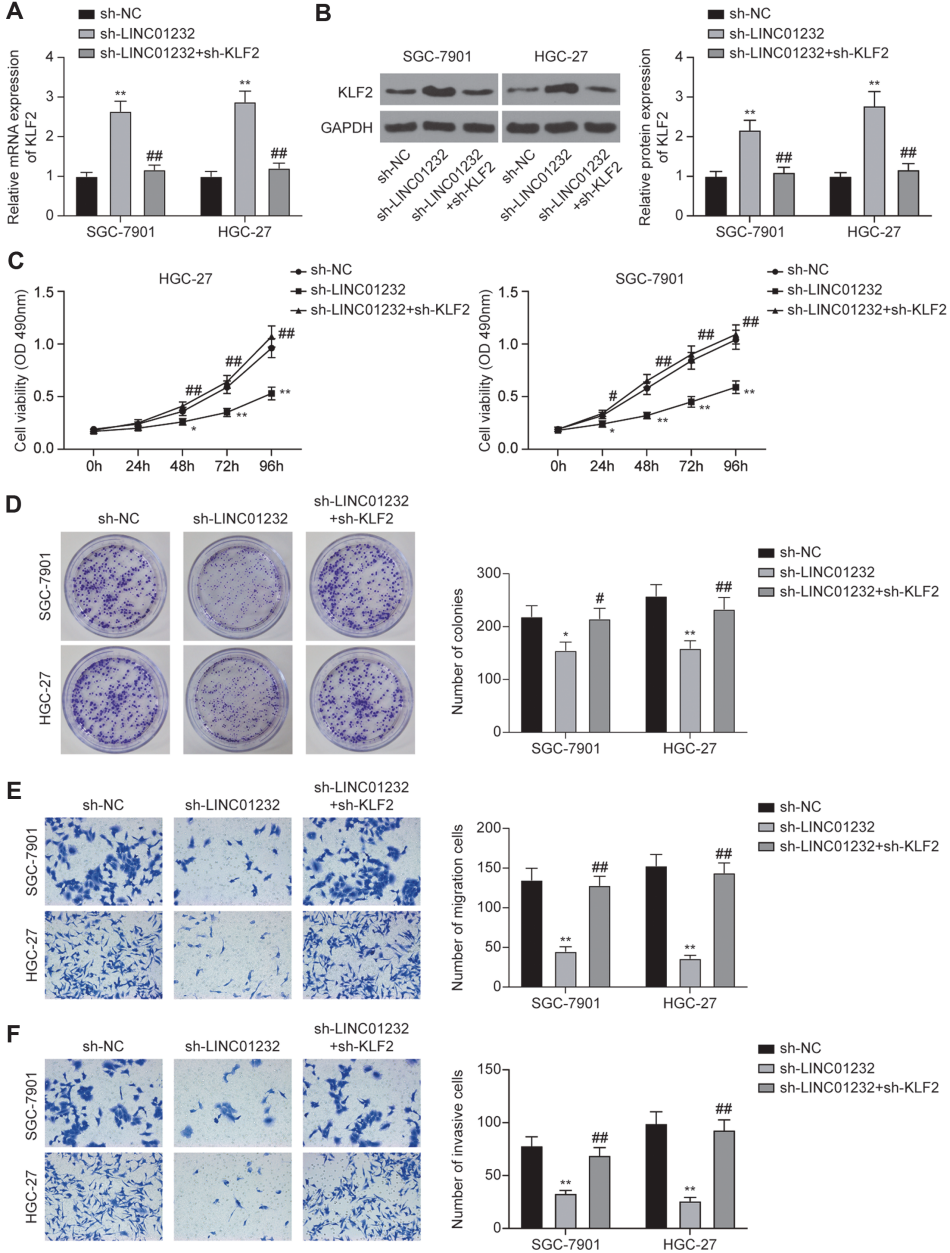
LINC01232 promotes gastric cancer proliferation through inhibiting KLF2. **A**: QRT-PCR was used to detect the mRNA expression of KLF2 in SGC-7901 and HGC-27 cells after transfection with sh-NC, sh-LINC01232 or sh- LINC01232+sh-KLF2. **B**: Western blot was used to detect the protein expression of KLF2 in SGC-7901 and HGC-27 cells after transfection with sh-NC, sh-LINC01232 or sh-LINC01232+sh-KLF2. **C**: MTT assay was used to detect the viability of SGC- 7901 and HGC-27 cells after transfection with sh-NC, sh-LINC01232 or sh-LINC01232+sh-KLF2. **D**: Colony formation assay was used to detect the proliferation of SGC-7901 and HGC-27 cells after transfection with sh-NC, sh-LINC01232 or sh- LINC01232+sh-KLF2. **E, F**: Transwell assay was used to detect the migration and invasion abilities of SGC-7901 and HGC-27 cells after transfection with sh-NC, sh-LINC01232 or sh-LINC01232+sh-KLF2. Values are mean ± SD. *p < 0.05, **p < 0.01 vs. sh-NC group; ^#^*p* < 0.05, ^##^*p* < 0.01 vs. sh-LINC01232 group.

To further clarify the functions of LINC01232 and KLF2 in the progression of gastric cancer, the expression of KLF2 in SGC-7901 and HGC-27 cells was knocked down on the basis of LINC01232 silencing. QRT-PCR (Fig. 4A) and western blot (Fig. 4B) results showed that the mRNA and protein expression of KLF2 was enhanced after LINC01232 knockdown, and KLF2 expression was decreased after KLF2 knockdown.

Compared with Sh-NC group, the cell viability (Fig. 4C), colony formation (Fig. 4D), migration (Fig. 4E) and invasion (Fig. 4F) were significantly reduced in sh-LINC01232 group (*p* < 0.05, respectively). Meanwhile, the knockdown of KLF2 could reverse the regulatory effect of LINC01232 in the proliferative ability of gastric cancer cells. These results demonstrated that the inhibition of KLF2 is essential to the tumor-promoting effect of LINC01232.

## Discussion

Gastric cancer is one of the most common cancers in the world, with risk factors that include *Helicobacter pylori* infection, age, high salt intake, and diets low in fruit and vegetables [15]. Early gastric cancer has no obvious symptoms, but with progression of the disease, dyspepsia and other symptoms of stomach discomfort gradually appear. However, the prognosis of advanced gastric cancer is poor, and the average 5-year survival rate is less than 20% [16]. Due to the poor prognosis and increasing incidence of gastric cancer, it is of great clinical significance to elucidate the mechanisms and screen biomarkers for early diagnosis of gastric cancer. Tumor genesis and development is a multi-factor and multi-stage process, which is influenced by many oncogenes, tumor suppressor genes and metastasis-related genes [17]. The expression of these genes is regulated not only at the transcriptional level, but also at the post-transcriptional level [18]. Non-coding RNAs (ncRNAs) are key components of the transcriptome and play important roles in both normal biological activity and pathological processes, such as viral infection and tumorigenesis [19]. Previous studies have demonstrated that LINC01232 exerts an oncogenic role in numerous types of tumors [8, 9]. In this study, we found that the expression of LINC01232 was upregulated in STAD tissues and gastric cancer lines, indicating that LINC01232 may be a functional gene and a molecular target for clinical treatment of gastric cancer. To further explore the role of LINC01232 in the progression of gastric cancer, we knocked down the expression of LINC01232 in SGC-7901 and HGC-27 cells by shRNA, and found that silencing of LINC01232LINC01232 can inhibit the proliferation, migration, invasion and tumorigenesis of gastric cancer cells.

Emerging evidence suggests that LncRNAs influence transcription of downstream factors through regulating histone acetylation or methylation as well as DNA methylation or hydroxy methylation [20-22]. EZH2 is a histone methylation enzyme that plays an important role in tumor invasion and metastasis. It also interacts with lncRNAs to affect the transcription of downstream factors [23]. Through RPISeq, RIP and qRT-PCR assays, we found that LINC01232 can interact directly with EZH2, which could target tumor suppressors such as p21, p53, KLF2 and LATS2. As a member of KLFs family, KLF2 contains three Cys 2/His 2 zinc finger motifs in tandem by which they bind to the common DNA-binding regions of transcriptional target sequences [24]. QRT-PCR and ChIP assays indicated that LINC01232 inhibits the expression of KLF2 through H3K27me3 histone methylation. We conducted rescue experiments to further clarify the functions of LINC01232 and KLF2 in the progression of gastric cancer. On the basis of the silencing of LINC01232, the expression of KLF2 in SGC-7901 and HGC-27 cells was knocked out, and the results showed that the inhibition of KLF2 was essential for the tumor-promoting effect of LINC01232.

In conclusion, our study showed that LINC01232 exerts oncogenic activities in gastric cancer and LINC01232 knockdown could inhibit the proliferation of gastric cancer cells in vivo and in vitro. The possible mechanism is related to inhibition of the expression of KLF2 through H3K27me3 histone methylation. The regulation of LINC01232 may be an effective potential therapeutic and preventive approach for gastric cancer. However, in future studies, an in-depth study of relationship among LINC0123, LSD1 and LATS2 still needs to be performed.
